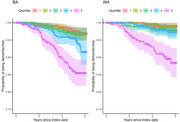# Higher ADRD machine learning phenotypic scores are associated with faster conversion to ADRD in Veterans without ICD ADRD diagnoses

**DOI:** 10.1002/alz70860_097753

**Published:** 2025-12-23

**Authors:** Debby W Tsuang, Karl Brown, Eugene Shao, Andrew Shutes‐David, Mark W. Logue, Qing Zeng

**Affiliations:** ^1^ University of Washington, Seattle, WA, USA; ^2^ VA Puget Sound, Seattle, WA, USA; ^3^ Geriatric Research, Education, and Clinical Center, Seattle, WA, USA; ^4^ George Washington University, Washington, DC, USA; ^5^ Seattle Institute for Biomedical and Clinical Research, Seattle, WA, USA; ^6^ Boston University Chobanian & Avedisian School of Medicine, Boston, MA, USA; ^7^ National Center for PTSD, VA Boston Healthcare System, Boston, MA, USA; ^8^ VA Washington DC Healthcare, Washington, DC, USA

## Abstract

**Background:**

Alzheimer's Disease and related dementias (ADRD) are under diagnosed and that under diagnosis if detrimental to patients, their families and the VA Health Care System. This crisis of under diagnosis exacerbates existing disparities in healthcare, disproportionately affect Black Americans (BAs) compared to White Americans (WAs). Using a machine learning (ML) model to assign ADRD‐risk scores may help to identify Veterans with undiagnosed ADRD and their risk of developing dementia in the near future.

**Method:**

We previously developed a ML model based on 850+ variables extracted from structured and unstructured data from the VHA's vast electronic health records, using a training sample of 20,000 BA and 20,000 WA Veterans. Kaplan‐Meier curves were calculated separately for race and ML score quintile group. A Cox‐proportions hazards model was used to obtain an estimate of the hazard ratio (HR) for the risk of developing ADRD.

**Result:**

The HR comparing the 75th to the 25th percentile of scores is estimated to be 2.20 (95% CI, 1.93‐2.51, *p* < 0.01). The Kaplan‐Meier plot showed differences in risk for converting to ADRD between races; for BAs, the curves of the highest two quintiles have visibly steeper slopes than those for other quintiles; for WAs, only the highest quintile have visibly steeper slopes than the other quintiles.

**Conclusion:**

Higher ML scores were associated with higher risk of developing ADRD in the years following the index date. BAs in our sample were at a higher risk for developing ADRD compared to their WA counterparts with the same score.